# Comparison of open and laparoscopic surgical techniques in colorectal cancer surgery: Early and late results

**DOI:** 10.1097/MD.0000000000049207

**Published:** 2026-06-05

**Authors:** Zülfi Zahidli, Uğur Topal, İshak Aydin, Yunus Kayci, Burak Yavuz, Ali Yildirim, Ahmet Gökhan Saritaş, İsmail Cem Eray, Ömer Alabaz

**Affiliations:** a Department of General Surgery, Hilvan Şehit Halit Şiltak State Hospital, Şanliurfa, Türkiye; b Department of General Surgery, Division of Surgical Oncology, Faculty of Medicine, Çukurova University, Adana, Türkiye; c Department of General Surgery, Faculty of Medicine, Çukurova University, Adana, Türkiye; d Department of General Surgery, Division of Gastroenterological Surgery, Adana City Training and Research Hospital, University of Health Sciences, Adana, Türkiye; e Department of General Surgery, Koç University Hospital, İstanbul, Türkiye.

**Keywords:** colon resection, colorectal cancer, laparoscopic colorectal surgery, laparoscopy

## Abstract

Colorectal cancer is the third most common malignancy worldwide and a leading cause of cancer-related mortality. Surgical resection remains the gold standard for curative treatment, while laparoscopic surgery offers potential advantages in postoperative recovery and complication rates. This study aimed to compare open and laparoscopic colorectal surgery in terms of perioperative, postoperative, pathological, oncological, and survival outcomes in a retrospective cohort. A retrospective analysis was conducted on 462 patients who underwent colorectal cancer surgery between January 2015 and June 2020. Patients were divided into open (n = 271) and laparoscopic (n = 191) groups. Demographic data, surgical characteristics, postoperative complications, pathological findings, and survival outcomes were analyzed. Survival analysis was performed using the Kaplan–Meier method, and comparisons were made using the log-rank test. Laparoscopic surgery was associated with significantly lower rates of surgical site infection (9.4% vs 31.0%, *P* < .001), intra-abdominal abscess (odds ratio = 7.17, *P* = .001), and shorter hospital stay (median 6 vs 10 days, *P* < .001). Early postoperative mortality was lower in the laparoscopic group (1.1% vs 4.8%, *P* = .026). Oncological parameters were comparable between groups, including resection margins and lymph node harvest. Distant metastasis was less frequent in the laparoscopic group (7.3% vs 16.3%, *P* = .022). Median overall survival was longer in the laparoscopic group (88.3 vs 73.2 months, *P* < .001); however, these crude survival differences are likely confounded by significant baseline imbalances between groups. Laparoscopic colorectal surgery is associated with improved short-term postoperative outcomes and comparable oncological parameters compared with open surgery. However, the groups were not balanced, with emergency cases and more advanced-stage disease predominantly present in the open surgery group; therefore, crude survival comparisons are subject to selection bias and should be interpreted cautiously. Laparoscopic surgery appears to be a safe and effective approach in selected patients with colorectal cancer.

## 1. Introduction

Colorectal cancer (CRC) is the third most common cancer worldwide and the second leading cause of cancer-related mortality.^[1]^ Early diagnosis is essential for improving outcomes, and current diagnostic work-up includes colonoscopy with biopsy and cross-sectional imaging.^[2]^ Despite advances in management, survival remains suboptimal in a considerable proportion of patients, particularly in advanced-stage disease.^[3]^

This study presents a retrospective, institutional comparison of open and laparoscopic colorectal cancer surgery in a selected real-world patient population. Therefore, outcomes should be interpreted in the context of inherent selection bias and baseline differences between treatment groups.

Surgical resection remains the cornerstone of curative treatment in colorectal cancer, and minimally invasive approaches have been increasingly adopted over the past decades due to their potential perioperative benefits.^[4,5]^

## 2. Methods

### 2.1. Methodology

This study was conducted after obtaining ethical approval from the Ethics Committee of Çukurova University on January 3, 2025 (Meeting No: 151). The study was designed as a retrospective, single-center observational cohort study and was carried out at a tertiary university hospital. The reporting of this study follows the Strengthening the Reporting of Observational Studies in Epidemiology guidelines for observational research.

A total of 462 patients who underwent either open or laparoscopic colorectal cancer surgery between January 2015 and June 2020 were included. Patients were categorized into open surgery (n = 271, 58.7%) and laparoscopic surgery (n = 191, 41.3%) groups. Twenty patients who required intraoperative conversion to open surgery were analyzed within the open group based on an intention-to-treat principle.

All patients older than 18 years who underwent elective or emergency colorectal surgery for malignancy were included. Data were retrospectively obtained from institutional records, pathology reports, imaging data, and follow-up files. Patients were staged according to the Tumor–Node–Metastasis classification system.

It is important to note that treatment allocation was not randomized and was determined by clinical decision-making. Significant baseline differences existed between groups, including a higher proportion of emergency cases and advanced-stage disease in the open surgery group, and a higher rate of rectal tumors and neoadjuvant therapy in the laparoscopic group. Therefore, potential selection bias must be acknowledged when interpreting comparative outcomes.

Evaluated variables included demographic characteristics, body mass index, American Society of Anesthesiologists score, tumor location, neoadjuvant therapy status, surgical characteristics, postoperative complications, histopathological findings (including lymphatic, vascular, and perineural invasion), pathological stage, and survival outcomes.

All patients underwent either complete mesocolic excision (CME) or total mesorectal excision (TME). Adjuvant therapy was administered according to standard oncological indications (stage III–IV disease). All deaths were classified as cancer-related based on hospital records and national registry data; however, early postoperative mortality may include multifactorial causes, which represents a potential limitation of this classification.

### 2.2. Statistical analysis

Statistical analyses were performed using IBM SPSS Statistics version 25.0. Normality was assessed using the Shapiro–Wilk and Kolmogorov–Smirnov tests. Continuous variables were expressed as mean ± standard deviation or median (interquartile range), as appropriate.

Group comparisons were performed using independent samples t-test or Mann–Whitney *U* test for continuous variables, and chi-square test for categorical variables.

A binomial logistic regression analysis was used to evaluate postoperative complications, with surgery type as the independent variable.

Survival analyses were conducted using unadjusted Kaplan–Meier estimates and log-rank tests, and results should be interpreted as exploratory comparisons rather than causal effects. Survival analyses were performed using the Kaplan–Meier method, and group comparisons were conducted using the log-rank test. A *P* value < 0.05 was considered statistically significant.

## 3. Results

A total of 462 patients were included in the study. Of these, 271 (58.7%) underwent open surgery and 191 (41.3%) underwent laparoscopic surgery.

The mean age of the patients was similar between the 2 groups: 61.6 ± 12.32 years in the open surgery group and 59.6 ± 12.45 years in the laparoscopic group (Table 1).

**Table 1:** Demographic and clinical characteristics of the patients.

Variable		Open (n = 271) 58.7%	Laparoscopic (n = 191) 41.3%	*P* value
Age (Mean ± SD) (Min–Max)		61.6 ± 12.32	59.6 ± 12.45	
Gender	Male	57.0% (166)	43% (125)	.358
	Female	61.4% (105)	38.6% (66)	
Urgency	Emergency	22.7% (57)	0	<.001
	Elective	77.3% (214)	100% (191)	
ASA Score	1	40.2% (109)	42.4% (81)	.088
	2	41.3% (112)	46.6% (89)	
	3	17.7% (87)	11.0% (21)	
	4	0.4% (1)	0	
BMI (Mean ± SD) (Min–Max)		25.9 ± 4.12	26.7 ± 4.85	.074
CEA ng/mL (95% CI: 0–3)(Mean ± SD) (Min–Max)		3.48 (1.8–7.8)	2.48 (1.48–5.65)	.015
Ca19.9 U/mL (95% CI: 0–37) (Mean ± SD) (Min–Max)		12.35 (5.9–29.2)	11.6 (4.1–22)	.067
Neoadjuvant Therapy	Received	19.9% (54)	42.4% (81)	<.001
	Not Received	80.1% (217)	57.6% (110)	
Tumor Localization	Cecum	20 (7.4)	12 (6.3%)	.001
	Ascending Colon	52 (19.2%)	13 (6.8%)	
	Hepatic Flexure	17 (6.3%)	4 (2.1%)	
	Transverse Colon	12 (4.4%)	5 (2.6%)	
	Splenic Flexure	13 (4.8%)	1 (0.5%)	
	Descending Colon	22 (8.1%)	7 (3.7%)	
	Sigmoid Colon	31 (11.4%)	32 (16.7%)	
	Rectosigmoid	25 (9.2%)	21 (11%)	
	Rectum	69 (25.5%)	95 (49.7%)	
	Multiple	10 (3.7%)	1 (0.6%)	

ASA = American Society of Anesthesiologists score, BMI = body mass index, CA 19-9 = Carbohydrate antigen 19-9, CEA = Carcinoembryonic antigen.

The need for intraoperative blood transfusion was more frequent in the laparoscopic group compared with the open surgery group (16.9% vs 11.9%, *P* = .048). Stapled anastomosis was used significantly more often in the laparoscopic group, whereas hand-sewn anastomosis was more frequently preferred in the open surgery group (*P* = .013). The rates of stoma formation were similar between the 2 groups (*P* = .806).

When operative time was evaluated, the median operative duration was 180 minutes (IQR: 150–200) in both groups. However, operative time was slightly longer in the laparoscopic group (*P* = .021). Intraoperative complication rates were low and comparable between groups (*P* = .175). Additional surgical procedures unrelated to the tumor were rare and similar between groups (*P* = .544) (Table 2).

**Table 2:** Intraoperative characteristics of the patients.

		Open (n, %)	Laparoscopic (n, %)	*P* value
Intraoperative Blood Transfusion	No	228 (88.1%)	159 (83.3%)	0.048
	Yes	43 (11.9%)	32 (16.9%)	
Anastomosis Technique	Hand-sewn	41 (15.1%)	12 (6.3%)	.013
	Stapled	204 (75.3%)	158 (82.7%)	
Stoma	No	162 (59.8%)	112 (58.6%)	.806
	Yes	109 (40.2%)	79 (41.4%)	
Operative time, min (median [IQR])		180 (150–200)	180 (150–200)	.021
Intraoperative Complication	No	96.3% (261)	98.4% (188)	.175
	Yes	3.7% (10)	1.6% (3)	
Additional Non Tumor Surgical Intervention	Cholecystectomy	1.5% (4)	1% (2)	.544
	Bladder Repair	0.4% (1)	0.5% (1)	
	Cystoscopy	0.4% (1)	0%	
	Splenectomy	0.7% (2)	0%	
	Adrenal Biopsy	0.4% (1)	0%	
	TAH BSO	0.4% (1)	0.5% (1)	
	Ureter Repair	1.5% (4)	0%	
	None	91.9% (249)	96.3% (184)	

Baseline characteristics demonstrated important differences between groups. Emergency surgery was observed only in the open surgery group (22.7% vs 0%), and stage IV disease was more frequent in the open group (17.1% vs 7.3%). In contrast, rectal tumor localization and neoadjuvant therapy were more common in the laparoscopic group. These differences represent significant selection bias and should be considered when interpreting all comparative outcomes.

No statistically significant differences were observed between groups in intra-abdominal abscess, ileus, anastomotic leakage, nonsurgical complications, postoperative bleeding, evisceration, readmission, or reoperation rates (all *P* > .05). Surgical site infection was lower in the laparoscopic group (*P* < .001). Postoperative hospital stay was also shorter in the laparoscopic group (*P* < .001). Early postoperative mortality was lower in the laparoscopic group (1.1% vs 4.8%, *P* = .026) (Table 3).

**Table 3:** Postoperative hospital stay, complications and early mortality according to surgical technique.

Outcome Category	Outcome	Open—Yes (%)	Open—No (%)	Laparoscopic—Yes (%)	Laparoscopic—No (%)	*P* value
Postoperative Complications	Intra-abdominal abscess	6.6 (18)	93.4 (253)	6.8 (13)	93.2 (178)	.913
	Wound infection	31.0 (84)	69.0 (187)	9.4 (18)	90.6 (173)	<.001
	Ileus	10 (27)	90 (244)	9.4 (18)	90.6 (173)	.847
	Anastomotic leakage	2.2 (6)	97.8 (265)	3.7 (7)	96.3 (184)	.353
	nonsurgical complications	7.4 (20)	92.6 (251)	7.9 (15)	92.1 (176)	.850
	Postoperative bleeding	1.5 (4)	98.5 (267)	2.6 (5)	97.4 (186)	.382
	Evisceration	4.1 (11)	95.9 (260)	1.6 (3)	98.4 (188)	.124
	Readmission	14 (38)	86 (233)	13.1 (25)	86.9 (166)	.773
	Reoperation	7.4 (20)	92.6 (251)	5.8 (11)	94.2 (180)	.493
	Early postoperative mortality	4.8 (13)	95.2 (258)	1.1 (2)	98.9 (188)	.026
Hospital Stay	Mean ± SD (days)	12.2 ± 8.06	—	7.08 ± 3.81	—	
	Median (days)	10.0	—	6.0	—	<.001

Binomial logistic regression analysis showed lower odds of surgical site infection and intra-abdominal abscess in the laparoscopic group compared with the open group (Table 4). The discrepancy between unadjusted comparisons and regression findings is attributable to differences in baseline risk profiles between groups, and these results should be interpreted as nonadjusted associations rather than causal effects.

**Table 4:** Binomial logistic regression analysis of postoperative complications between open and laparoscopic surgery (Ref: Open Surgery).

Complication	Estimate	SE	Z	*P*	OR
Wound infection	1.463	0.280	5.22	<.001	4.32
Intra-abdominal abscess	1.97	0.615	3.205	.001	7.17
Ileus	−0.141	0.316	−0.445	.657	0.87
Evisceration	0.975	0.658	1.48	.138	2.65
Gastroparesis	−18.3	4827	−0.004	.997	–
Obstruction	17.66	3487	0.005	.996	–
Anastomotic leakage	0.474	0.608	0.778	.436	1.61
Urinary fistula	−0.764	0.918	−0.832	.405	0.47
Urinary retention	0.215	0.634	0.339	.735	1.24
Urinary incontinence	16.97	3487	0.005	.996	–
Bleeding	0.919	0.807	1.14	.255	2.51
Readmission	0.003	0.275	0.013	.990	1.00

When oncological outcomes were evaluated (Table 5), radial and distal surgical margins and lymph node harvest were comparable between groups (*P* > .05). Vascular and perineural invasion rates were higher in the open surgery group (*P* = .043 and *P* = .001, respectively).

**Table 5:** Comparison of oncological outcomes, histopathological diagnoses, and tumor invasion characteristics according to surgical technique in patients operated for colorectal cancer.

Parameters	Open surgery	Laparoscopic surgery	*P* value
Radial Surgical Margin(Negative)	Rectum: 69 (100%) Colon: 197 (97.5%)	Rectum: 94 (98.9%) Colon: 96 (100%)	Rectum: 0.393 Colon: 0.491
Distal Surgical Margin (mm)	Rectum: 4 (2.3–5.3) Colon: 9.5 (5–16.3)	Rectum: 3.5 (2–5.5) Colon: 7 (5–10)	Rectum: 0.621 Colon: 0.699
Number of Harvested Lymph Nodes	Rectum: 9 (6–15) Colon: 19 (12–30)	Rektum: 10 (6–15) Colon: 19 (11–27)	Rectum: 0.697 Colon: 0.165
Number of Metastatic Lymph Nodes	Rectum: 0 (0–2) Colon: 1 (0–3)	Rectum: 0 (0–2) Colon: 0 (0–2)	Rectum: 0.513 Colon: 0.699
Histopathological Diagnosis			
Adenocarcinoma	266 (98.2%)	180 (94.2%)	.001
Mucinous Adenocarcinoma	51 (19.17%)	25 (13.08%)	—
Signet Ring Cell Carcinoma	0 (0%)	0.5 (1%)	—
Neuroendocrine Tumor	0 (0%)	3 (1.6%)	—
Pathological Complete Response	0 (0%)	7 (3.77%)	—
Lymphatic Invasion	Present: 229 (84.5%) Absent: 42 (15.5%)	Present: 148 (77.5%) Absent: 43 (22.5%)	.093
Vascular Invasion	Present: 225 (83%) Absent: 46 (17%)	Present: 142 (79.4%) Absent: 49 (20.6%)	.043
Perineural Invasion	Present: 168 (62%) Absent: 103 (38%)	Present: 87 (45.5%) Absent: 104 (54.5%)	.001
Tumor Perforation	Present: 10 (3.7%) Absent: 261 (96.3%)	Present:2 (1%) Absent: 189 (99%)	.200

In terms of long-term oncological outcomes (Table 6), peritoneal carcinomatosis, local recurrence, and para-aortic recurrence rates were similar between groups (*P* > .05). Distant organ metastasis was more frequent in the open surgery group (16.3% vs 7.3%, *P* = .022**).** However, these findings are likely influenced by baseline imbalances, particularly higher-stage and emergency cases in the open group, and should not be interpreted as treatment-related effects.

**Table 6:** Postoperative long-term oncological outcomes according to surgical technique.

Outcome category	Outcome	Open—Yes (%)	Open—No (%)	Laparoscopic—Yes (%)	Laparoscopic—No (%)	*P* value
Long-term oncological outcomes	Peritoneal carcinomatosis	4.1% (11)	—	1.6% (3)	—	.124
	Local recurrence	3.0% (8)	—	1.6% (3)	—	.338
	Paraaortic recurrence	1.5% (4)	—	0.5% (1)	—	.330
	Distant organ metastasis	16.3% (44)	—	7.3% (14)	—	.022

Regarding Tumor–Node–Metastasis classification staging, stage IV disease was significantly more frequent in the open group, while early-stage disease was slightly more common in the laparoscopic group.

The unadjusted mean survival time was 73.19 months in the open surgery group and 88.25 months in the laparoscopic group (*P* < .001). Survival analyses were not adjusted for confounding variables such as stage, urgency, or neoadjuvant therapy; therefore, observed differences likely reflect baseline group imbalances rather than treatment effect. Stage-specific survival differences were observed at certain time points, but overall 5-year survival differences were not consistent across stages (Table 7).

**Table 7:** Distribution of TNM stages and survival outcomes according to surgical technique.

Category		Open surgery	Laparoscopic surgery	*P* value
TNM stages	Stage 1	47 (17.3%)	35 (18.3%)	.001
	Stage 2	81 (30.1%)	63 (33.0%)	
	Stage 3	97 (36.1%)	66 (34.6%)	
	Stage 4*	46 (17.1%)	14 (7.3%)	
	Total	271 (100%)	191 (100%)	
Overall survival	Mean survival (months)	73.19 (67.04–79.33)	88.25 (81.84–94.65)	<.001
	1-year survival	84.1% (n = 228)	95.3% (n = 181)	<.001
	3-year survival	63.1% (n = 171)	77.5% (n = 148)	<.001
	5-year survival	52.3% (n = 136)	67.0% (n = 122)	.002
Stage-specific 1-year survival	Stage 1	90.2%	97.1%	.227
	Stage 2	90.1%	93.7%	.447
	Stage 3	85.6%	97.0%	.016
	Stage 4	65.2%	85.7%	.143
Stage-specific 3-year survival	Stage 1	80.5%	91.4%	.177
	Stage 2	76.5%	82.5%	.379
	Stage 3	62.9%	71.2%	.270
	Stage 4	21.7%	50.0%	.040
Stage-specific 5-year survival	Stage 1	78.9%	84.4%	.561
	Stage 2	62.5%	74.6%	.133
	Stage 3	50.5%	63.1%	.118
	Stage 4	11.4%	7.7%	.705

* *P* < .05 compared with the laparoscopic group.

## 4. Discussion

The choice of surgical approach in colorectal cancer surgery has important implications for perioperative outcomes and recovery. However, this study represents a retrospective and nonbalanced cohort, and therefore all comparisons between open and laparoscopic surgery should be interpreted with caution due to significant selection bias.

In crude analyses, laparoscopic surgery appeared to be associated with lower early postoperative mortality and improved 1-, 3-, and 5-year survival outcomes. However, these differences may be influenced by baseline imbalances between the groups, particularly the higher proportion of emergency cases and advanced-stage disease in the open surgery group. After stage-based stratification, survival differences between the groups were largely attenuated, suggesting that disease severity rather than surgical approach was the primary determinant of long-term outcomes.

In terms of perioperative outcomes, laparoscopic surgery was associated with reduced surgical site infection and intra-abdominal abscess rates, shorter hospital stay and lower incisional hernia formation. These findings are consistent with the known benefits of minimally invasive surgery.

Laparoscopic colorectal surgery is widely accepted for its reduced surgical trauma and faster postoperative recovery.^[4,6,7]^ From a technical perspective, proper oncologic dissection, particularly in right colectomy, requires strict adherence to embryological planes and vascular anatomy, as emphasized in recent anatomical and surgical standardization studies.^[5]^ These principles are essential to ensure oncological adequacy in minimally invasive colectomy.

Operative time was longer in the laparoscopic group, consistent with prior literature. Prolonged operative duration has been associated with increased postoperative morbidity in some studies, although this relationship is influenced by case complexity and learning curve effects.^[8–12]^

After the landmark randomized trials including Clinical Outcomes of Surgical Therapy trial,^[13]^ Conventional versus Laparoscopic-Assisted Surgery in Colorectal Cancer trial,^[14]^ and Colon Cancer Laparoscopic or Open Resection trial,^[15]^ and the Cochrane review by Kuhry et al.,^[16]^ laparoscopic surgery has been established as oncologically equivalent to open surgery in appropriately selected patients. More recent evidence, including a systematic review and meta-analysis of laparoscopic and robotic surgery in older patients, further supports the safety and feasibility of minimally invasive approaches across broader patient populations.^[17]^

Regarding oncologic outcomes, lymph node harvest and margin status were comparable between groups in our study. This suggests adequate oncologic resection in both approaches, consistent with prior literature emphasizing the importance of CME or TME principles. Importantly, recent evidence has shown that the survival benefit attributed to CME is most evident only after adjustment for stage and preoperative treatment variables.^[18]^ This further highlights the importance of controlling for baseline differences when interpreting survival outcomes in retrospective studies.

Distant metastasis was more frequently observed in the open surgery group; however, this is likely confounded by the higher proportion of advanced-stage and emergency cases in this group rather than a direct effect of surgical technique.

Although crude survival rates appeared higher in the laparoscopic group, these findings should not be interpreted as evidence of oncologic superiority. Instead, they reflect the imbalance in baseline characteristics between the groups.

### 4.1. Key message

“In this retrospective and nonbalanced cohort, laparoscopy was associated with better short-term recovery and similar pathological adequacy; long-term comparisons remain limited by selection bias.”

This interpretation is consistent with major randomized controlled trials and meta-analyses demonstrating oncological equivalence between laparoscopic and open colorectal surgery in appropriately selected patients.

Some retrospective studies have reported improved survival with laparoscopic approaches^[19,20]^; however, these results likely reflect selection effects and tumor biology rather than a direct causal benefit of surgical technique.

### 4.2. Limitations

The main limitations of this study include its retrospective design, single-center nature, and significant baseline imbalance between groups, particularly regarding emergency surgery, tumor stage, and neoadjuvant therapy distribution. In addition, absence of propensity-score matching or multivariable survival adjustment limits the strength of causal inference.

## 5. Conclusions

In this retrospective, single-center observational study, laparoscopic colorectal surgery was associated with lower rates of selected short-term postoperative complications, including surgical site infection, intra-abdominal abscess, and incisional hernia, as well as shorter hospital stay. In contrast, operative time was longer in the laparoscopic group compared with open surgery.

Oncological outcomes, including resection margins, lymph node harvest, and recurrence patterns, were comparable between laparoscopic and open surgery groups. Similarly, when baseline stage differences were taken into account, the 2 groups were found to be comparable.

Given the retrospective design and substantial baseline imbalance between groups, particularly differences in emergency surgery rates and tumor stage distribution—these results should be interpreted as associative findings rather than causal evidence.

Overall, laparoscopic surgery appears to be a safe and effective surgical option in appropriately selected colorectal cancer patients, offering improved short-term recovery while maintaining oncological adequacy. However, robust conclusions regarding long-term survival require prospective, multicenter studies with appropriate risk adjustment and standardized reporting according to observational study guidelines.

## Author contributions

**Conceptualization:** Zülfi Zahidli, Uğur Topal, İshak Aydin, Ömer Alabaz.

**Data curation:** Zülfi Zahidli, Uğur Topal, İshak Aydin, Yunus Kayci, Burak Yavuz, Ali Yildirim, Ahmet Gökhan Saritaş, İsmail Cem Eray.

**Formal analysis:** Zülfi Zahidli, Uğur Topal, Burak Yavuz.

**Investigation:** Yunus Kayci, Ömer Alabaz.

**Methodology:** Yunus Kayci, Ahmet Gökhan Saritaş.

**Project administration:** İsmail Cem Eray, Ömer Alabaz.

**Resources:** Zülfi Zahidli, Ali Yildirim.

**Software:** Zülfi Zahidli, İsmail Cem Eray.

**Supervision:** Zülfi Zahidli, İshak Aydin, Ahmet Gökhan Saritaş, Ömer Alabaz.

**Validation:** Ali Yildirim, Ömer Alabaz.

**Visualization:** Zülfi Zahidli, Burak Yavuz.

**Writing – original draft:** Zülfi Zahidli.

**Writing – review & editing:** Zülfi Zahidli, Uğur Topal, İshak Aydin, Yunus Kayci, Burak Yavuz, Ali Yildirim, Ahmet Gökhan Saritaş, İsmail Cem Eray, Ömer Alabaz.
